# Congenital Laryngeal Hemangioma: A Case Report of a Rare Presentation

**DOI:** 10.7759/cureus.34814

**Published:** 2023-02-09

**Authors:** Peter Appiah-Thompson, Nana Andoh M Hanson, Kofi Quansah, Emmanuel Kobina Mesi Edzie, Jimah Bashiru Babatunde

**Affiliations:** 1 Surgery/Otolaryngology, School of Medical Sciences, University of Cape Coast, Cape Coast, GHA; 2 Surgery/Otolaryngology, Cape Coast Teaching Hospital, Cape Coast, GHA; 3 Radiology, School of Medical Sciences, University of Cape Coast, Cape Coast, GHA

**Keywords:** congenital haemangioma, vascular tumours, vascular malformations, vascular anomalies, supraglottic, subglottic, capillary, cavernous, infantile laryngeal haemangioma

## Abstract

Pediatric vascular anomalies are classified into vascular malformations and vascular tumors. While vascular malformations are generally anomalous vessels, vascular tumors arise from endothelial cells characterized by abnormal proliferation. Vascular tumors, also called hemangiomas, are subdivided into infantile and congenital hemangiomas. The differentiation of these anomalies can be challenging, and immunohistochemical staining is often employed for this purpose. The GLUT-1 (erythrocyte-type glucose transporter protein) stain is positive for the infantile type.

Hemangiomas are usually found in the head and neck region. Their occurrence in the laryngeal region in infants tends to manifest in the subglottic region. Hemangiomas in the larynx mostly do not cause any symptoms until they are large enough to cause dyspnea, stridor, or hoarseness of voice. They are mostly treated in infants with propranolol or surgical excision.

We report a case of an eight-day-old female infant who presented with a mass that recurrently protruded out of the mouth when she cried. The mass stopped protruding out of the mouth when the baby became restless, had respiratory distress, and refused feeds. Endoscopy of the pharynx and larynx showed a pedunculated hemorrhagic mass attached by a stalk to the left arytenoid. With cautery, the stalk of the lesion was severed from its attachment. The baby was discharged on the fourth postoperative day and histology reported a cavernous hemangioma.

Seven months after the surgery, the baby is growing normally. Yearly follow-up endoscopies have been scheduled to evaluate for recurrence or residual disease.

## Introduction

Pediatric vascular anomalies are classified into vascular malformations and vascular tumors. The vascular malformations generally comprise anomalous vessels, while the vascular tumors arise from the endothelial cells characterized by abnormal proliferation. The vascular malformations, though present at birth, grow slowly and mostly become enlarged due to hormonal activity (such as at menarche or during pregnancy) or in the setting of trauma or surgery. Vascular tumors, also called hemangiomas, are subdivided into infantile and congenital hemangiomas. The differentiation of these anomalies can be very difficult, and immunohistochemical staining is helpful for their differentiation. The GLUT-1 (erythrocyte-type glucose transporter protein) stain is positive for the infantile type. Infantile hemangiomas are generally not present at birth, and, if they do occur, they only appear as a reddened or pale area where the lesion subsequently appears. The congenital type, which involves high-flow vascular tumors, manifests at birth. The congenital type can involute over a period of a year or may persist [1,2].

Hemangiomas are the most common tumors of infancy and are mostly found in the head and neck region [3]. These lesions tend to be more common in children as compared to adults [4]. The usual locations of these tumors are the subglottic region for the infantile laryngeal type and the supraglottic region for the adult laryngeal type [5]. They rarely transform into malignancies even in adults [6].

In children, the presence of congenital laryngeal hemangiomas may cause stridor or dyspnea due to their subglottic location in most cases [7]. The diagnosis of these lesions is usually made in newborns based on the observation of the aforementioned clinical features. The clinical presentation of these lesions tends to be dependent on their size. In adults, they might not cause any symptoms until they are incidentally noted on endoscopy, e.g., during direct laryngoscopy [7]. CT scans or MRIs would be able to reveal the exact locations and sizes of these lesions. Even though a detailed history, examination, flexible nasolaryngoscopy, and CT scanning may point to the lesion as being a vascular anomaly, histology is needed to confirm the diagnosis of hemangioma.

## Case presentation

An eight-day-old baby girl was referred to our tertiary healthcare facility, with a history of intermittent protrusion of a fleshy swelling out of the mouth since birth. The baby had a normal cry but was noted to have difficulty feeding when the swelling returned to the throat. The examination showed a pink, healthy baby with a reddish mass that periodically protruded out of the mouth when she cried, as shown in Video 1.

**Video 1:** Reddish mass protruding out of the mouth of the baby when she cries

A tentative diagnosis of a pharyngeal mass was made with a differential diagnosis of a lingual thyroid gland. An initial ultrasound of the neck found a normal thyroid gland. A nasogastric tube was blindly passed with caution and tube-feeding was started since breastfeeding was difficult for the baby. During admission, the baby developed a fever, which was managed with IV cefazolin and IV paracetamol. While awaiting a CT scan report, the baby became restless and started crying excessively, and the mass no longer protruded from the oral cavity. Emergency examination of the pharynx and larynx under anesthesia was then done after successful endotracheal intubation.

The intraoperative findings were as follows: i) a pedunculated reddish mass with a pedicle attached to the superior surface of the left arytenoid region with the mass completely buried in the esophagus, and ii) a normal oral cavity, pharynx, and laryngeal structures (apart from the left arytenoid).

**Figure 1:**
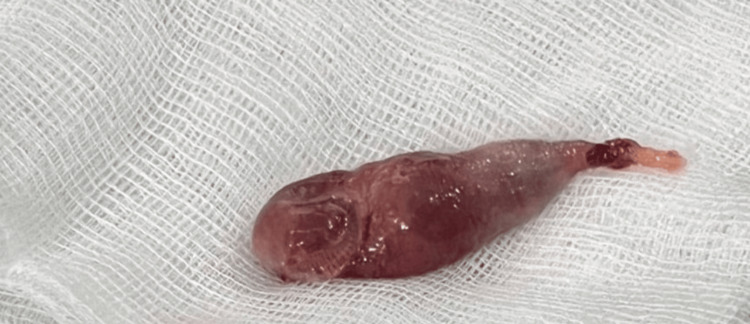
Reddish laryngeal mass after excision

The reddish lesion was then teased out of the esophagus with forceps and the pedicle was severed with diathermy under direct laryngoscopy with a Chevalier Jackson laryngoscope. The specimen, i.e. the lesion as seen in Figure 1, was then sent for histopathology.

**Figure 2:**
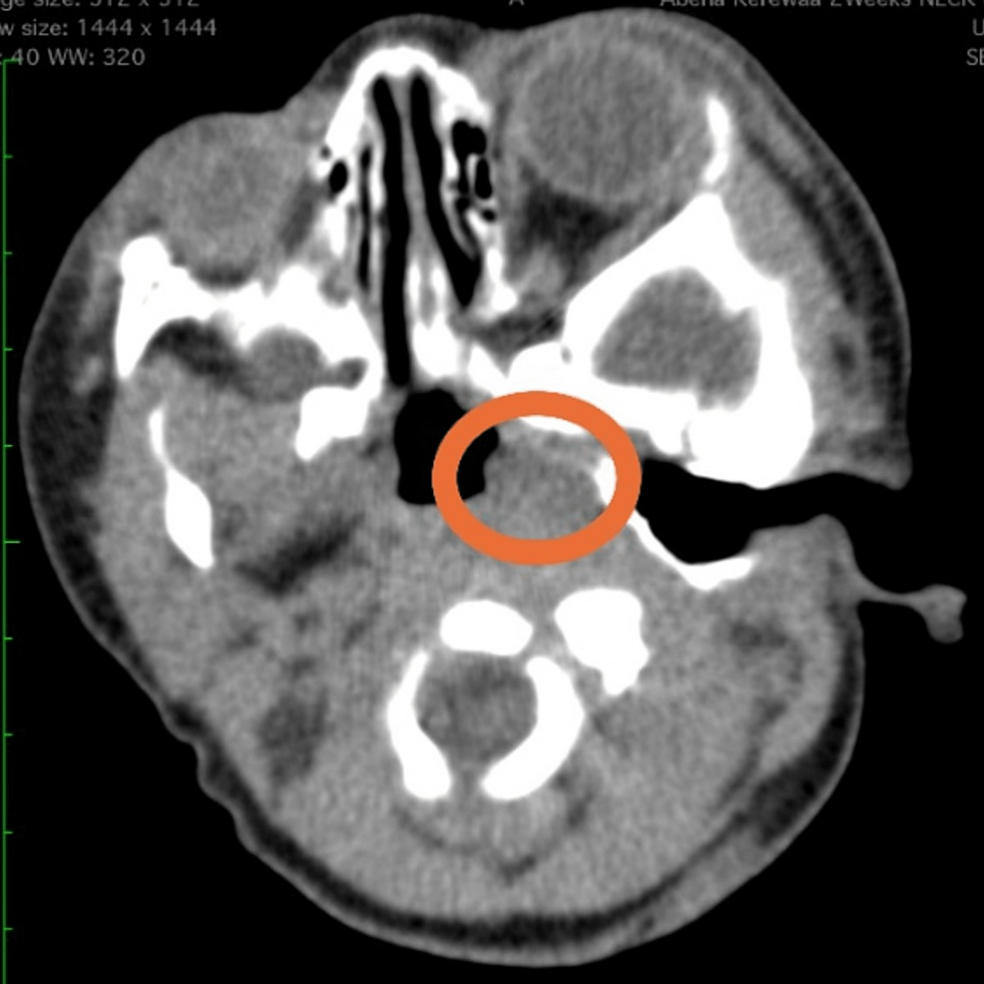
Axial CT scan of the paranasal sinuses and pharynx showing a mass in the left half of the nasopharynx (encircled) CT: computed tomography

The baby recovered well after surgery and was discharged on the fourth postoperative day. The CT scan, which was received after the surgery, showed a poorly differentiated mass located in the left half of the nasopharynx, as shown in Figure 2. 

Histology reported the following findings: sections show proliferation of thick and thin-sized blood vessels in the dermis of varying sizes admixed with lobules of fat. The overlying epithelium was ulcerated. Diagnosis: cavernous hemangioma.

Seven months after surgery, the baby continues to grow very well.

## Discussion

Vascular anomalies have been grouped into vascular malformations and vascular tumors. Vascular malformations are present at birth in most cases but grow slowly and are challenged to grow more by trauma and hormones. Vascular tumors, which result from endothelial hyperplasia, are classified into the infantile type and the congenital type. The infantile type is differentiated from all other vascular anomalies by the positive GLUT-1 staining [1,2], which, unfortunately, is not available in our country.

Laryngeal hemangiomas are mostly found in the subglottic region in infants with the adult type being found attached to supraglottic structures such as epiglottis, aryepiglottic folds, arytenoids, and false and true vocal cords [5,8]. In our case, unlike the mostly reported location, the lesion was attached by a stalk to the left arytenoid in a baby.

Laryngeal hemangiomas occur in different forms, i.e., cavernous, capillary, or mixed types. The capillary type tends to have larger vascular channels with deeper infiltration into the submucosa while the cavernous types are well-circumscribed and less infiltrative [8]. Our case was a cavernous type.

Management of these lesions depends on their size. Small lesions may be treated with observation and follow-up. Larger lesions causing symptoms may require intralesional injections of corticosteroids, carbon dioxide laser or potassium-titanyl-phosphate laser, or surgical excision [9]. Generally, due to the risk of residual disease and recurrence, laryngeal lesions must be followed up with endoscopy and biopsy [10]. We hope to follow up with our patient yearly with flexible laryngoscopy to avoid missing any residual disease or recurrence.Hemangiomas in children have been shown to respond to propranolol as well [3], though in this case, due to the bulky nature of the lesion and the negative impact it was having on the baby’s health, a complete surgical excision was carried out to prevent death. Awaiting improvement for days with propranolol, as reported in other cases [11], was not feasible in our case as we could have lost the baby due to respiratory distress. It has been argued that radical surgical excision of hemangioma is necessary when the lesion is circumscribed and attached only to the submucosal layer. This is believed to prevent recurrence and possible later complications as the structures of phonation are mostly spared [8]. The lesion in our case was excised without injuring the underlying tissues.

## Conclusions

Congenital laryngeal hemangioma may or may not present any significant clinical features. Babies born with masses in the oral cavity should undergo careful endoscopy to evaluate for such lesions and clinicians should maintain a high index of suspicion for laryngeal hemangioma. Surgical excision is an effective method of treatment for laryngeal hemangiomas causing respiratory distress or other obstructive symptoms.
